# Angiogenesis in NSCLC: is vessel co-option the trunk that sustains the branches?

**DOI:** 10.18632/oncotarget.7794

**Published:** 2016-02-29

**Authors:** Ana Luísa Coelho, Mónica Patrícia Gomes, Raquel Jorge Catarino, Christian Rolfo, Agostinho Marques Lopes, Rui Manuel Medeiros, António Manuel Araújo

**Affiliations:** ^1^ Instituto Português de Oncologia, Molecular Oncology Group, Porto, Portugal; ^2^ Faculdade de Medicina, University of Porto, Porto, Portugal; ^3^ Instituto de Ciências Biomédicas Abel Salazar, University of Porto, Porto, Portugal; ^4^ Phase I, Early Clinical Trials Unit, Antwerp University Hospital, Edegem, Belgium; ^5^ Centre of Oncological Research (CORE), Antwerp University, Edegem, Belgium; ^6^ Centro Hospitalar de S. João, Pulmonology Department, Porto, Portugal; ^7^ Liga Portuguesa Contra o Cancro (NRNorte), Research Department, Porto, Portugal; ^8^ Centro Hospitalar do Porto, Medical Oncology Department, Porto, Portugal

**Keywords:** NSCLC, angiogenesis, anti-angiogenic strategies, vessel co-option, angiopoietin-2

## Abstract

The critical role of angiogenesis in tumor development makes its inhibition a valuable new approach in therapy, rapidly making anti-angiogenesis a major focus in research. While the VEGF/VEGFR pathway is the main target of the approved anti-angiogenic molecules in NSCLC treatment, the results obtained are still modest, especially due to resistance mechanisms. Accumulating scientific data show that vessel co-option is an alternative mechanism to angiogenesis during tumor development in well-vascularized organs such as the lungs, where tumor cells highjack the existing vasculature to obtain its blood supply in a non-angiogenic fashion. This can explain the low/lack of response to current anti-angiogenic strategies. The same principle applies to lung metastases of other primary tumors. The exact mechanisms of vessel co-option need to be further elucidated, but it is known that the co-opted vessels regress by the action of Angiopoietin-2 (Ang-2), a vessel destabilizing cytokine expressed by the endothelial cells of the pre-existing mature vessels. In the absence of VEGF, vessel regression leads to tumor cell loss and hypoxia, with a subsequent switch to a neoangiogenic phenotype by the remaining tumor cells. Unravelling the vessel co-option mechanisms and involved players may be fruitful for numerous reasons, and the particularities of this form of vascularization should be carefully considered when planning anti-angiogenic interventions or designing clinical trials for this purpose. In view of the current knowledge, rationale for therapeutic approaches of dual inhibition of Ang-2 and VEGF are swiftly gaining strength and may serve as a launchpad to more successful NSCLC anti-vascular treatments.

## INTRODUCTION

Cancer is a major health issue, constituting the second leading cause of death worldwide and expected to surpass heart diseases as the leading cause of death in the next few years [1, 2]. In 2013, the incidence of cancer cases worldwide was 14.9 million, with 8.2 million cancer-related deaths. Lung cancer was the most common incident form of cancer, with an estimated 1.8 million new cases having deaths that exceeded those from any other type of malignancy worldwide, accounting for nearly one in five deaths (1.6 msillion deaths in total) [2]. Most lung cancers (~85%) are non-small cell lung cancers (NSCLC) which are divided according to two major histologic subtypes: the non-squamous carcinomas (mainly adenocarcinomas) and the squamous-cell carcinomas [3]. The parenchyma and the stroma are the two almost-indistinguishable compartments that compose the NSCLC and build up the tumor microenvironment [4]. The stromal cells contribute to the development and expression of certain cancer hallmark capabilities, defined by Hanahan and Weinberg in 2011 [5]. Among these, angiogenesis assumes major importance, since rate-limiting steps in tumor progressions include gaining access to the host vascular system and the generation of a tumor blood supply to obtain oxygen and nutrients, growth factors and hormones [6].

## ANGIOGENESIS AND CANCER

While the identification of massive vascularization in tumors dates back to 1863 [7] and the importance of tumor angiogenesis has been recognised since 1908 [8], it was only through the work of Folkman in the early 1970s that the scientific community acknowledge angiogenesis as a potential target to inhibit cancer progression [9–12]. The therapeutic potential of anti-angiogenic strategies boosted this field of research, placing angiogenesis as one of the major hubs of current cancer research.

It is now widely accepted that most tumors and metastases originate as small avascular structures which must induce the development of new blood vessels from pre-existing ones in order to grow beyond a minimum size of 2-3 mm^3^ [6, 13]. To achieve this, tumors undergo an angiogenic switch, disrupting the equilibrium between pro and anti-angiogenic regulators and favouring pro-angiogenic mechanisms. Signalling molecules induce quiescent endothelial cells to continuously sprout from existing blood vessels, thereby forming new vessels that help to sustain expanding neoplastic growth [6, 14, 15], according to the conventional model of angiogenesis known as angiogenic sprout [16].

Decades of research investigating the molecular basis of angiogenesis led to the discovery of a number of angiogenic molecules that promote tumor angiogenesis [15]. Of all the identified angiogenic pathways, the most critical appears to be the one involving the VEGF family and their receptors (VEGFR1-2-3) [17–19], although a number of other important molecules and their receptors have also proven to work in combination with VEGF/VEGFR signalling in tumor angiogenesis [19]. These include the fibroblast growth factor receptors (FGFRs) family and their ligands, particularly FGF1 and FGF2, that induce the proliferation and migration of endothelial cells [20]; as well as the platelet-derived growth factor receptors (PDGFRs) and their ligands (PDGFs) that, either alone or in combination with FGF and VEGF, are associated with tumor vascularization in malignant disease, including NSCLC [21, 22] and the Ang-Tie-2 system [19, 22]. Ever since the identification of VEGF as the first endothelium-acting specific cytokine in 1983 [13, 23, 24], its overexpression has been found in several human tumors, including NSCLC [25–29]. More recently, scientists are gaining a better understanding of the many functions of this molecule in the tumor angiogenic process [29]: it triggers multiple signalling networks that enhance endothelial cell proliferation and survival, increases migration and invasion of endothelial cells, increases vascular permeability of existing vessels, and enhances chemotaxis and mobilization of bone marrow derived endothelial progenitor cells (EPCs) into the peripheral circulation [30, 31].

The growing acknowledgment of VEGF's key role in tumor angiogenesis has made it an attractive target for therapeutic intervention in cancer. The VEGF pathway is a promising avenue in research that aims to uncover more effective, targeted anti-angiogenic strategies [23, 32]. The extensive investigation in this field has led to the study of several anti-angiogenic agents, including monoclonal antibodies to block VEGF and its receptor VEGFR2 and VEGFR tyrosine kinase inhibitors (TKIs) [33].

## ANTI-ANGIOGENIC THERAPY AND LUNG CANCER

From the multitude of potential therapeutic options that target angiogenesis in NSCLC, [34] (Table 1), there are currently three anti-angiogenic compounds approved by EMA for the treatment of NSCLC. Bevacizumab, an anti-VEGF monoclonal antibody that blocks the binding of VEGF to its high-affinity receptors, was the first angiogenic inhibitor to complete clinical development, showing clinical benefit in patients with metastatic colorectal cancer when combined with chemotherapy [31, 33]. It was approved in 2006 for the treatment of advanced non-squamous NSCLC in the first line setting in combination with chemotherapy [29]. In 2014, ramucirumab, a fully humanized monoclonal antibody that targets angiogenesis by specifically binding to VEGFR-2 with higher affinity than its natural ligand VEGF [35], was approved for the treatment of patients with metastatic NSCLC in second line setting, in combination with docetaxel [36]. In the same year, nintedanib, an oral medication that can simultaneously inhibit triple angiokinase, VEGFR, platelet-derived growth factor receptors (PDGFR), and fibroblast growth factor receptors (FGFR) signalling pathways, was approved to be used in combination with docetaxel in patients with locally advanced, metastatic, or locally recurrent NSCLC adenocarcinoma, after first-line chemotherapy [37]. There are also other potential agents that are under clinical evaluation, whether that be in the clinical trial stage or currently waiting for approval for treatment of metastatic or recurrent NSCLC [38, 39].

**Table 1:** Angiogenesis inhibitors in non-small cell lung cancer (NSCLC)

Approved		
Drug	Target	Indication
Bevacizumab	VEGF	First-line treatment of nonsquamous NSCLC with CT
Nintedanib	VEGFR 2, FGFR 1-3, PDGFRα and β TKI	Second-line treatment of adenocarcinoma NSCLC with CT
Ramucirumab	VEGFR-2	Second-line treatment of NSCLC with CT
**On clinical trials or not approved**		
**Drug**	**Target**	
Vandetanib	VEGFRs, EGFR, and RET	
Sunitinib	VEGFRs, PDGFRs, KIT, FLT3, CSF-1R, and RET	
Aflibercept	VEGF	
Sorafenib	VEGFR, PDGFRs, FGFR, KIT, and RAF	
Motesanib	VEGFRs, PDGFRs, and KIT	
Pazopanib	VEGFRs, PDGFRs, FGFR, and KIT	
Cediranib	VEGFRs	
Cabozantinib	VEGFR, RET, and MET	
Axitinib	VEGFRs, PDGFRs, and KIT	

CT - chemotherapy; VEGF - vascular endothelial growth factor; VEGFR - vascular endothelial growth factor receptor; FGFR - fibroblast growth factor receptor; PDGFR - platelet-derived growth factor receptor; KIT - stem cell factor receptor; FLT3 - Fms-like tyrosine kinase-3; CSF-1R - colony stimulating factor receptor; RET - glial cell-line derived neurotrophic factor receptor; MET - met proto-oncogene;

In spite of the impressive clinical efficacy of bevacizumab, ramucirumab, and nintedanib in various cancer treatment settings, the results were relatively modest and limited [40]. In addition, the clinical use of VEGF/VEGFR blockers as anti- angiogenic therapy for patients with advanced NSCLC has been more challenging than anticipated by the preclinical experiments in which long-term benefit of VEGF/VEGFR inhibition was achieved [41]. Anti-angiogenic agents are usually given to all patients for the approved indications; in a high fraction of these patients, however, the tumor is intrinsically refractory to the anti-angiogenic therapy and the disease progresses ceaselessly [42]. Moreover, when there is no intrinsic resistance, acquired resistance to therapy can rapidly occur and limit the efficacy of the anti-angiogenic treatments [41, 43], and the clinical benefit of prolonging cancer patients survival with advanced disease becomes limited, often in the order of weeks or months [16, 44].

Tumor resistance to the anti-angiogenic therapies (whether intrinsic or acquired), represents a significant problem faced in routine clinical practice. The mechanisms underlying the response to these therapies are far from being clearly understood, further fuelling this active field of research [43]. Preclinical investigations have shed some light on the subject, and although different authors propose escape ways from angiogenic inhibitors that are somewhat distinct, some key features appear to be consensual among most of them; these features are likely to be involved in primary and acquired resistance and deserve consideration [16, 18, 41–43, 45]. One of such features is invasive (or metastatic) co-option of normal quiescent vessels without requisite of angiogenesis.

## VESSEL CO-OPTION AND LUNG CANCER GROWTH

It is widely accepted that tumor progression is heavily dependent on angiogenesis. Much less understood, is the concept that angiogenesis is necessary for a tumor to become larger than a few millimetres and become clinically detectable, as some research has shown that angiogenesis is not always a pre-requisite for tumor growth [46]. Hence, one possibility for anti-angiogenic therapy resistance is that some primary and metastatic tumors are non-angiogenic, meaning that these tumors do not need angiogenic sprout to obtain an efficient blood supply [47]. Rather, the tumors use alternative vascularization mechanisms. For example, in vessel-dense tissues, the most likely route is hijacking the pre-existing normal blood vessels [42, 44, 48], and more aggressive tumors can undergo vasculogenic mimicry, a process by which tumor cells dedifferentiate to an endothelial phenotype forming structures that provide tumour cells with a secondary circulation system independently of angiogenesis [49].

When tumors arise in well-vascularized organs, their growth will rely on the invasion of host tissue. Enhancement of invasion and metastasis facilitates access to normal tissue vasculature, and cancer cells stay in close contact with the surface of blood vessels [39, 50, 51]. This allows tumor cells to grow and migrate along quiescent normal vessels and take their oxygen and essential nutrients without obligate neovascularization, in a process known as vessel (or vascular) co-option [42, 43, 49]. These non-angiogenic tumors are a separate group of fast-growing malignancies with little apoptosis and very efficient mitochondrial metabolism [52]. This seems to be the case of tumors arising in the lungs, liver, and brain, areas where this form of vascularization appears to assume a major role [31, 50, 51, 53, 54]. This is also true for tumor metastasis that occurs through lymph and blood vessels and outgrow mostly in these vessel-dense organs [55–58].

In recent years, research related to angiogenesis has been massive; but on the contrary, there is a scarcity of research focusing on tumors that escape pathways of classical angiogenesis and use vessel co-option as an alternative blood supply for tumor growth. This has led to a dearth in information regarding the mechanisms and players involved in that process.

The first insights into the relationship between vessel co-option and lung cancer were made by Pezzella and co-workers, who described NSCLC that grew without morphological evidence of neoangiogenesis but with signs of normal tissue vessel exploitation [59]. They characterized these tumors as having an alveolar pattern, with tumor cell nests filling the alveolar spaces without destruction of the lung parenchyma. The only vessels evident in these tumors appeared to belong to the trapped alveolar septa [59]. Moreover, patients with alveolar pattern tumors presented a worse survival rate than their angiogenic counterparts. Later, when investigating the possible role of microvessel count in NSCLC as a potential marker of disease prognosis, Offersen and colleagues [60] identified the same special vascular pattern in 17 out of 35 NSCLC samples, thus confirming the description of Pezzella's group. Their observations led them to the hypothesis that these alveolar tumors are nonangiogenic and invasive and exploited the pre-existing vascular beds. They also noted that some tumors exhibited only the alveolar pattern while other tumors presented a mixed alveolar pattern consisting of both alveolar and angiogenic features [60]. There was no correlation, however, between angiogenic or vessel co-option status and disease aggressiveness.

Taking into account the NSCLC growth patterns, Nia Sardari *et al*. suggested a modification of Pezzella's classification according to morphological features, based on the biological properties of the tumor-lung interface, which is the region where the tumor expands and the tumor-stroma interactions are more active and homogeneous [61]. According to them, NSCLCs can be classified as having a destructive growth pattern (angiogenic growth pattern), papillary growth pattern (with preservation of the alveolar structure of the lung parenchyma at the interface with co-option of alveolar blood vessels with formation of stromal stalks and subsequent angiogenesis), and alveolar growth pattern (preservation of the alveolar structure of lung parenchyma with co-option of septal blood vessels and without evidence of new stroma formation at the interface). Moreover, they suggested that, in NSCLC, a low degree of ongoing angiogenesis is predictive of poorer prognosis [61, 62].

The hypothesis of co-option by lung metastases, which are often the main cause of death in many solid malignancies, was also proposed by Pezzella's group back in the 1990's. They observed that, regardless of the angiogenic status of the primary breast carcinomas, they could relapse as nonangiogenic tumors in the lungs. This was also true for lung metastases of human renal and colorectal carcinomas [56, 63, 64]. In a very recent study, Szabo and co-workers used cell lines from six different solid tumors, and showed that lung metastases vascularize by co-opting the pulmonary microvasculature. The investigated cell lines incorporated the pre-existing host tissue capillaries within the alveolar walls, striping the epithelium from these co-opted alveolar walls [57]. Once there, the metastases expand as the malignant cells spread from one alveolar space to another. Their work not only shed some light on the mechanisms underlying this phenomenon, but it also raised some questions surrounding the biology of the nonangiogenic tumors, further advocating the need for additional exploration in this subject.

## VESSEL CO-OPTION AND ANGIOPOIETIN-2

In the lungs, the normal co-opted vessels trapped in the tumor can be very effective because they allow for more efficient tumor growth by exploiting the highly regular vascular network of the lungs and progressively filling the empty alveolar spaces [46]. Regardless of the efficacy of vessel co-option in sustaining tumor growth, the quiescent blood vessels co-opted by tumors suffer extreme changes over time [65]. While there is still debate if this due to a host defence mechanism against tumor development [47] or whether dependence on the survival of endothelial cells (ECs) [50], there is little doubt on the subsequent alterations observed. First, in the centre of the tumor, there is widespread regression of the co-opted vessels associated with the regression of the EC, turning it progressively hypoxic, with subsequent massive tumor cell loss [13, 32], followed by a robust *de novo* angiogenesis at the outer rim of the tumor, that rescues the remaining tumor cells in a later stage [13, 31].

**Figure 1:**
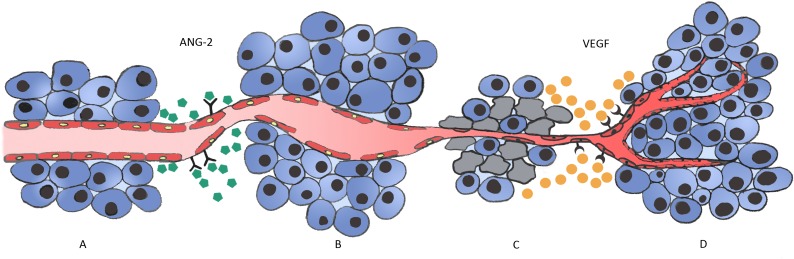
Vessel co-option and Ang-2 regulation in cancer development in vessel dense tissues **A**. In well vascularized organs, such as the lung, tumor cells grow and migrate along quiescent normal vessels (vessel co-option). **B.** Over time, tumor cells induce extreme changes in the co-opted vessels and ECs start to express Ang-2, leading to vascular disruption and vessel regression. **C**. Regression of the co-opted vessel associated with regression of the ECs generates a hypoxic core in the tumor centre, with massive tumor cell loss. This triggers the angiogenic switch, with the remaining tumor cells expressing high amounts of VEGF. **D**. VEGF expression induces a robust angiogenic response that ultimately rescues the tumor and allows its growth and progression.

The key regulator in the regression of the initially co-opted blood vessels appears to be Angiopoietin-2 (Ang-2) [49, 53, 66], a cytokine that belongs to the Angiopoietins family, an important class of angiogenic molecules. It is a natural ligand of the endothelial tyrosine kinase-receptor, Tie-2, primarily synthetized and secreted by ECs at sites of vascular remodelling, like tumors, in a tightly regulated fashion [66–68]. Ang-2 is overexpressed in a number of tumors including NSCLC [69, 70], and there is also evidence that it is deeply involved in lung metastases homing and progression [71, 72]. Experimental evidence supports the notion that, soon after vessel co-option, host vessels start to express high levels of Ang-2 that acts through an endogenous autocrine loop mechanism that is context dependent [73, 74]. When it binds to its Tie-2 receptor, it functions as a vessel-destabilizing molecule that converts mature vessels to a tenuous and plastic state by inducing loosening of endothelial cell interactions with pericytes and smooth muscle cells, leading to the loss of vascular integrity and increased vascular permeability. The ECs of such destabilized vessels can be prone to two fates, depending on the local cytokine milieu [74, 75]. In the presence of VEGF, these cells will respond to the proliferating signals induced by the pro-angiogenic molecule and will migrate or proliferate, triggering a sprouting angiogenesis [13, 66, 70, 73, 76, 77]. In the absence of VEGF, however, the expression of Ang-2 causes irreversible loss of vascular structures [76,78] with marked regression of the co-opted vessels, as is the case when tumors co-opt pre-existing vessels [77]. This is due to the fact that, without the pericytes coverage, the ECs of the Ang-2-unstable vessels will die [79] in a very similar fashion to what happens with primitive vessels during development [74]. This generates the hypoxic core and the apoptotic tumor cell loss observed in nonangiogenic tumors [47, 76], that presumably act as the initial stimulus for the molecular changes that culminate in VEGF expression by the remaining tumor cells and in neoangiogenesis [69], mediated both by VEGF and Ang-2 [47] (Figure 1).

Not surprisingly, the discovery of the role of Ang-2 in tumor progression led to the suggestion that its inhibition could translate into clinically meaningful responses, opening the door to multiple approaches that have been used to experimentally inhibit Ang-2 as well as explore its effects on angiogenesis and tumor growth [80–82]. Pre-clinical models revealed that Ang-2 inhibition reduces the growth of a broad range of tumors. Although some of the results were modest, some revealed to be very promising and there is now a robust pipeline of drugs targeting the Ang/Tie-2 system in different clinical trials phases (Table 2) [67, 78, 83]. Furthermore, with Ang-2 being required to render endothelium responsive to VEGF and with both molecules contributing to tumor angiogenesis and metastases [84, 85], there seems to be a more encouraging response to the straightforward question of whether co-targeting of both ligands in a bispecific manner would improve the outcomes of current anti-angiogenic therapies [80, 83, 86–88].

**Table 2:** Ang-1/Ang-2 and Tie inhibitors in development for NSCLC

Drug	Target	Studies
Regorafenib	VEGFRs, PDGFRs, FGFR, RET, Kit, B-Raf and Tie-2	NCT01187615
Trebananib Fc fusion peptibody	Ang-1 and Ang-2	NCT01666977, EudraCT 2011-001111-31
Foretinib	VEGFRs, PDGFR β, FLT3, MET, and Tie-2	NCT01068587
MGCD265	VEGFRs, MET, and Tie-2	NCT02544633, EudraCT 2015-002070-21
AMG 780 Fully human anti Ang-1/2 mAb	Ang-1 and Ang-2	NCT01137552

VEGFR - vascular endothelial growth factor receptor; PDGFR - platelet-derived growth factor receptor; FGFR - fibroblast growth factor receptor; RET - glial cell-line derived neurotrophic factor receptor; KIT - stem cell factor receptor; Tie - endothelial tyrosine kinase-receptor; FLT3 - Fms-like tyrosine kinase-3; B-Raf - serine/threonine-protein kinase;

## VESSEL CO-OPTION AND CLINICAL IMPLICATIONS

The ability to identify tumors that make vascular co-option their primary source of blood supply does not envisage an easy task, hence why few strategies have been used to achieve this goal [48]. Research in the field has been scarce, especially when compared to the angiogenic field that has largely overshadowed alternative blood sources for tumor development. Moreover, much of the research has been performed in cell lines or murine models and only a few in human tissues [48]. While the findings are limited so far, what has been discovered highly advocates for unravelling the vessel co-option mechanisms and involved players. The precise identification of tumors that preferentially use this route to support growth and the factors driving them to switch from this to an angiogenic pattern may be crucial to delineate future cancer treatments for two main reasons. The first is that vascular co-option may represent a clever strategy by which tumors partly evade and resist conventional anti-angiogenic treatments [89]. Even if a treatment like bevacizumab is effective against one angiogenic factor such as VEGF, the therapy can still fail if this factor is not important for the endothelium in that given tumor, as appears to be the case in tumors that co-opt pre-existing vessels in NSCLC [45]. In these cases, vessel co-option may serve as a pathologic biomarker for selecting potentially nonresponsive patients [43]. There is also evidence that in some nonangiogenic tumors, cancer cells adapt by migrating more aggressively into normal tissue [42]; and when anti-angiogenic treatments are used indiscriminately, they may contribute to the selection of clones of nonangiogenic cells that will progress with a more aggressive behaviour [89, 90]. These features should be carefully considered when planning anti-angiogenic therapeutic interventions, suggesting the need for tailor-made treatments against such tumors.

Secondly, anti-angiogenic compounds do not affect incorporated pre-existent vasculature or matured tumor vasculature, making targeting existing vessels on which the tumor growth relies, an attractive approach to accomplish tumor regression [91]. This is also of primordial importance in cases of metastases that establish in well-vascularized organs, since vessel co-option may constitute their primary feeding option [57]. Moreover, it can be speculated that in earlier stages of the tumor, the interval that mediates Ang-2 overexpression, co-opted vessels regression, and *de novo* angiogenesis seems to be the perfect therapeutic window for intervention using a dual-pronged approach with Ang-2 and VEGF blockers rather than in more advanced stages of the disease. This issue should be addressed by investigators developing pre-clinical/clinical trials of drugs that target angiogenesis or envisage tumor arrest by anti-angiogenic strategies.

## CONCLUSIONS

Anti-angiogenic strategies focusing on VEGF/VEGFR in combination with chemotherapy marked a milestone in the field of cancer treatment, including NSCLC. However, a relevant number of patients are unresponsive or refractory to anti-angiogenic treatments. Some tumors obviate the need to generate angiogenesis by co-opting host mature vessels and growing along them, using them as blood sources. Vessel co-option is a mechanism that may help explain the limited success of anti-angiogenic therapy in these patients in an adjuvant setting.

Thus far, the only growth factors proven to be associated with vessel co-option are VEGF and Ang-2. This lack of information is likely due to the limited number of studies examining this subject. Ang-2 seems to have a particularly critical role in the process, but is also an extremely laborious study topic due to the complexity of its functions and regulation, which are both highly cell context dependent.

Tumors that grow in non-angiogenic fashions through exploitation of pre-existing vessels are non-responsive to anti-angiogenic molecules and raise a number of concerns in terms of treatment. First, little is known about the modifications a neoplastic cell must go through in order to co-opt a blood vessel, which is a huge obstacle for strategies that aim to interfere with this step in tumor progression. Second, once the tumor is committed to vascular co-option pathway, an effective way of blocking tumor progression would be to target existing tumor vasculature; this would require the availability of tumor-vessel specific targeting agents, however, and the few candidates that have been identified so far have failed to prove their clinical efficacy.

All of these concerns reinforce the need for better understanding of the mechanisms and molecular players underlying vessel co-option during tumor development within the proper biologic context. This would not only explore more assertive cancer treatments and help with the identification of tumors where vessel co-option is the growth support (instead of angiogenesis), but could also help identify patients who may be nonresponsive to current anti-angiogenic treatments. Additionally, it could open doors to novel areas of NSCLC research at both the molecular and microanatomical level.
